# Diagnostic criteria for multiple sclerosis: 2010 Revisions to the McDonald criteria

**DOI:** 10.1002/ana.22366

**Published:** 2011-02

**Authors:** Chris H Polman, Stephen C Reingold, Brenda Banwell, Michel Clanet, Jeffrey A Cohen, Massimo Filippi, Kazuo Fujihara, Eva Havrdova, Michael Hutchinson, Ludwig Kappos, Fred D Lublin, Xavier Montalban, Paul O'Connor, Magnhild Sandberg-Wollheim, Alan J Thompson, Emmanuelle Waubant, Brian Weinshenker, Jerry S Wolinsky

**Affiliations:** 1 Department of Neurology, Free UniversityAmsterdam, the Netherlands; 2 Scientific and Clinical Review Associates LLCNew York, NY; 3 Division of Neurology, Hospital for Sick ChildrenToronto, Ontario, Canada; 4 Department of Neurosciences, University Hospital CenterToulouse, France; 5 Mellen Center for Multiple Sclerosis Treatment and Research, Cleveland Clinical FoundationCleveland, OH; 6 Neuroimaging Research Unit, Division of Neuroscience, Scientific Institute and University Hospital San RaffaeleMilan, Italy; 7 Department of Multiple Sclerosis Therapeutics, Tohoku University Graduate School of MedicineSendai, Japan; 8 Department of Neurology, First Faculty of Medicine, Charles UniversityPrague, Czech Republic; 9 St Vincent's University HospitalElm Park, Dublin, Ireland; 10 Departments of Neurology and Biomedicine, University Hospital, KantonsspitalBasel, Switzerland; 11 Corrine Goldsmith Dickinson Center for Multiple Sclerosis, Department of Neurology, Mount Sinai School of MedicineNew York, NY; 12 Clinical Neuroimmunology Unit, Multiple Sclerosis Center of Catalonia, University Hospital Vall d'HebronBarcelona, Spain; 13 Division of Neurology, St. Michael's Hospital, University of TorontoToronto, Ontario, Canada; 14 Department of Neurology, University HospitalLund, Sweden; 15 University College London Institute of NeurologyUnited Kingdom; 16 Multiple Sclerosis Center, University of CaliforniaSan Francisco, CA; 17 Department of Neurology, Mayo ClinicRochester, MN; 18 Department of Neurology, University of Texas Health Sciences CenterHouston, TX

## Abstract

New evidence and consensus has led to further revision of the McDonald Criteria for diagnosis of multiple sclerosis. The use of imaging for demonstration of dissemination of central nervous system lesions in space and time has been simplified, and in some circumstances dissemination in space and time can be established by a single scan. These revisions simplify the Criteria, preserve their diagnostic sensitivity and specificity, address their applicability across populations, and may allow earlier diagnosis and more uniform and widespread use. Ann Neurol 2011

Diagnostic criteria for multiple sclerosis (MS) include clinical and paraclinical laboratory assessments^1^,^2^ emphasizing the need to demonstrate dissemination of lesions in space (DIS) and time (DIT) and to exclude alternative diagnoses. Although the diagnosis can be made on clinical grounds alone, magnetic resonance imaging (MRI) of the central nervous system (CNS) can support, supplement, or even replace some clinical criteria,^3^–^9^ as most recently emphasized by the so-called McDonald Criteria of the International Panel on Diagnosis of MS.^8^,^9^ The McDonald Criteria have resulted in earlier diagnosis of MS with a high degree of both specificity and sensitivity,^10^–^13^ allowing for better counseling of patients and earlier treatment.

Since the revision of the McDonald Criteria in 2005, new data and consensus have pointed to the need for their simplification to improve their comprehension and utility and for evaluating their appropriateness in populations that differ from the largely Western Caucasian adult populations from which the Criteria were derived. In May 2010 in Dublin, Ireland, the International Panel on Diagnosis of MS (the Panel) met for a third time to examine requirements for demonstrating DIS and DIT and to focus on application of the McDonald Criteria in pediatric, Asian, and Latin American populations.

## Considerations Related to Revisions to the McDonald Criteria

The Panel reviewed published research related to the diagnosis of MS and to the original and revised McDonald Criteria, gathered from literature searches of English language publications containing the terms *multiple sclerosis* and *diagnosis*, and from specific recommendations of relevant papers by Panel members. The Panel concluded that most recent research supports the utility of the McDonald Criteria in a typical adult Caucasian population seen in MS centers, despite only limited research and practical experience in general neurology practice populations.

In its discussions, the Panel stressed that the McDonald Criteria should only be applied in those patients who present with a typical clinically isolated syndrome (CIS) suggestive of MS or symptoms consistent with a CNS inflammatory demyelinating disease, because the development and validation of the Criteria have been limited to patients with such presentations. CIS presentations can be monofocal or multifocal, and typically involve the optic nerve, brainstem/cerebellum, spinal cord, or cerebral hemispheres.

In applying the McDonald Criteria, it remains imperative that alternative diagnoses are considered and excluded. Differential diagnosis in MS has been the subject of previous data- and consensus-driven recommendations that point to common and less common alternative diagnoses for MS and identify clinical and paraclinical red flags that should signal particular diagnostic caution.^14^,^15^ In its current review, the Panel focused specifically on the often-problematic differential diagnosis for MS of neuromyelitis optica (NMO) and NMO spectrum disorders. There is increasing evidence of relapsing CNS demyelinating disease characterized by involvement of optic nerves (unilateral or bilateral optic neuritis), often severe myelopathy with MRI evidence of longitudinally extensive spinal cord lesions, often normal brain MRI (or with abnormalities atypical for MS), and serum aquaporin-4 (AQP4) autoantibodies.^16^,^17^ There was agreement that this phenotype should be separated from typical MS because of different clinical course, prognosis, and underlying pathophysiology and poor response to some available MS disease-modifying therapies.^18^ The Panel recommends that this disorder should be carefully considered in the differential diagnosis of all patients presenting clinical and MRI features that are strongly suggestive of NMO or NMO spectrum disorder, especially if (1) myelopathy is associated with MRI-detected spinal cord lesions longer than 3 spinal segments and primarily involving the central part of the spinal cord on axial sections; (2) optic neuritis is bilateral and severe or associated with a swollen optic nerve or chiasm lesion or an altitudinal scotoma; and (3) intractable hiccough or nausea/vomiting is present for >2 days with evidence of a periaqueductal medullary lesion on MRI.^19^,^20^ In patients with such features, AQP4 serum testing should be used to help make a differential diagnosis between NMO and MS to help avoid misdiagnosis and to guide treatment.

Correct interpretation of symptoms and signs is a fundamental prerequisite for diagnosis.^21^ The Panel considered again what constitutes an attack (relapse, exacerbation) and defined this as patient-reported symptoms or objectively observed signs typical of an acute inflammatory demyelinating event in the CNS, current or historical, with duration of at least 24 hours, in the absence of fever or infection. Although a new attack should be documented by contemporaneous neurological examination, in the appropriate context, some historical events with symptoms and evolution characteristic for MS, but for which no objective neurological findings are documented, can provide reasonable evidence of a prior demyelinating event. Reports of paroxysmal symptoms (historical or current) should, however, consist of multiple episodes occurring over not less than 24 hours. There was consensus among the Panel members that before a definite diagnosis of MS can be made, at least 1 attack must be corroborated by findings on neurological examination, visual evoked potential (VEP) response in patients reporting prior visual disturbance, or MRI consistent with demyelination in the area of the CNS implicated in the historical report of neurological symptoms.

The Panel concluded that the underlying concepts of the original (2001) and revised (2005) McDonald Criteria^8^,^9^ are still valid, including the possibility of establishing a diagnosis of MS based on objective demonstration of dissemination of lesions in both space and time on clinical grounds alone or by careful and standardized integration of clinical and MRI findings. However, the Panel now recommends key changes in the McDonald Criteria related to the use and interpretation of imaging criteria for DIS and DIT as articulated by the recently published work from the MAGNIMS research group.^22^–^24^ Such changes are likely to further increase diagnostic sensitivity without compromising specificity, while simplifying the requirements for demonstration of both DIS and DIT, with fewer required MRI examinations. The Panel also makes specific recommendations for application of the McDonald Criteria in pediatric and in Asian and Latin American populations.

### Recommended Modifications to the McDonald Criteria: The 2010 Revisions

#### Magnetic Resonance Imaging Criteria for DIS

In past versions of the McDonald Criteria, DIS demonstrated by MRI was based on the Barkhof/Tintoré criteria.^4^,^6^ Despite having good sensitivity and specificity, these criteria have been difficult to apply consistently by nonimaging specialists.^25^,^26^ The European MAGNIMS multicenter collaborative research network, which studies MRI in MS, compared the Barkhof/Tintoré criteria for DIS^4^,^6^ with simplified criteria developed by Swanton and colleagues.^22^,^27^ In the MAGNIMS work, DIS can be demonstrated with at least 1 T2 lesion in at least 2 of 4 locations considered characteristic for MS and as specified in the original McDonald Criteria (juxtacortical, periventricular, infratentorial, and spinal cord), with lesions within the symptomatic region excluded in patients with brainstem or spinal cord syndromes. In 282 CIS patients, the Swanton-based DIS criteria were shown to be simpler and slightly more sensitive than the original McDonald Criteria for DIS, without compromising specificity and accuracy.^22^ The Panel accepted these MAGNIMS DIS Criteria, which can simplify the diagnostic process for MS while preserving specificity and improving sensitivity (Table 1).

**TABLE 1:** 2010 McDonald MRI Criteria for Demonstration of DIS

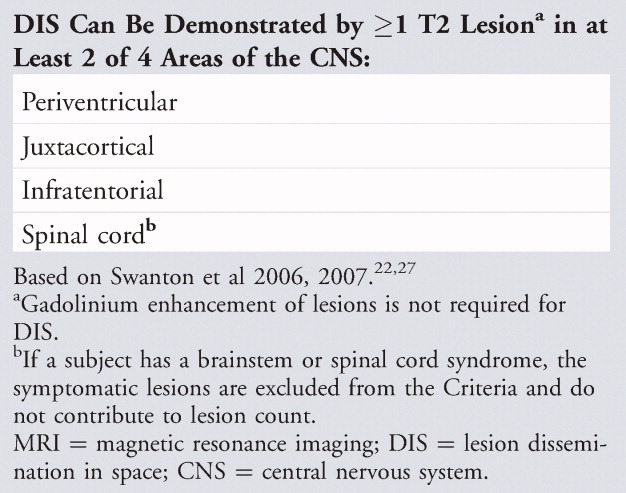

#### Magnetic Resonance Imaging Criteria for DIT

The 2005 revision of the McDonald Criteria simplified the MRI evidence required for DIT, basing it on the appearance of a new T2 lesion on a scan compared to a reference or baseline scan performed at least 30 days after the onset of the initial clinical event.^9^ In clinical practice, however, there is reason not to postpone a first MRI until after 30 days of clinical onset, which would result in an extra MRI scan to confirm a diagnosis. Abandoning the requirement for an extra reference MRI after 30 days does not compromise specificity,^28^ and therefore the Panel, in its current revision of the McDonald Criteria, allows a new T2 lesion to establish DIT irrespective of the timing of the baseline MRI.

More recently, the MAGNIMS group confirmed earlier studies^29^,^30^ by showing that, in patients with typical CIS, a single brain MRI study that demonstrates DIS and both asymptomatic gadolinium-enhancing and non-enhancing lesions is highly specific for predicting early development of clinically definite MS (CDMS) and reliably substitutes for prior imaging criteria for DIT.^23^,^24^ After review of these data, the Panel accepted that the presence of both gadolinium-enhancing and nonenhancing lesions on the baseline MRI can substitute for a follow-up scan to confirm DIT (Table 2), as long as it can be reliably determined that the gadolinium-enhancing lesion is not due to non-MS pathology.

**TABLE 2:** 2010 McDonald MRI Criteria for Demonstration of DIT

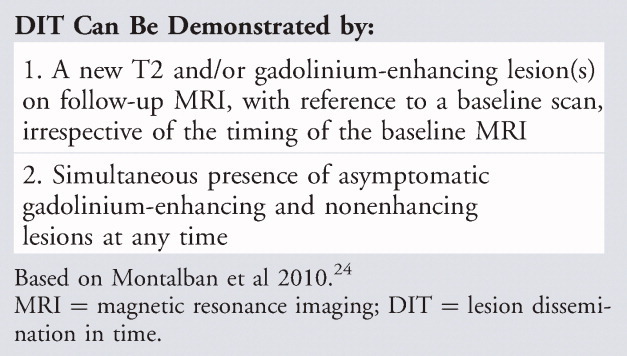

By using the recommended simplified MAGNIMS criteria to demonstrate DIS^22^ and allowing DIT to be demonstrated by a scan containing both enhancing and nonenhancing lesions in regions of the CNS typical for MS,^23^ a diagnosis of MS can be made in some CIS patients based on a single MRI.^24^ The Panel felt this is justified because it simplifies the diagnostic process without reducing accuracy. However, a new clinical event or serial imaging to show a new enhancing or T2 lesion will still be required to establish DIT in those patients who do not have both gadolinium-enhancing and nonenhancing lesions on their baseline MRI.

#### The Value of Cerebrospinal Fluid Findings in Diagnosis

The Panel reaffirmed that positive cerebrospinal fluid (CSF) findings (elevated immunoglobulin G [IgG] index or 2 or more oligoclonal bands) can be important to support the inflammatory demyelinating nature of the underlying condition, to evaluate alternative diagnoses, and to predict CDMS.^15^,^31^ In the 2001 and 2005 McDonald Criteria, a positive CSF finding could be used to reduce the MRI requirements for reaching DIS criteria (requiring only 2 or more MRI-detected lesions consistent with MS if the CSF was positive).^8^,^9^ However, when applying the simplified MAGNIMS imaging criteria for DIS and DIT,^24^ the Panel believes that even further liberalizing MRI requirements in CSF-positive patients is not appropriate, as CSF status was not evaluated for its contribution to the MAGNIMS criteria for DIS and DIT.^22^,^24^ Prospective studies using widely available standardized techniques and the most sensitive methods of detection of oligoclonal bands in the CSF together with the new imaging requirements are needed to confirm the additional diagnostic value of CSF.^32^,^33^

#### Making a Diagnosis of Primary Progressive Multiple Sclerosis

In 2005, the Panel recommended revising the McDonald Criteria for diagnosis of primary progressive multiple sclerosis (PPMS) to require, in addition to 1 year of disease progression, 2 of the following 3 findings: positive brain MRI (9 T2 lesions or 4 or more T2 lesions with positive VEP); positive spinal cord MRI (2 focal T2 lesions); or positive CSF. These criteria reflected the special role of both CSF examination and spinal cord MRI in PPMS, have been found to be practical and are generally well accepted by the neurological community,^34^ and have been used as inclusion criteria for PPMS clinical trials.^35^ To harmonize MRI criteria within the diagnostic criteria for all forms of MS, while recognizing the special diagnostic needs for PPMS, the Panel recommends that the McDonald Criteria requirement of fulfilling 2 of 3 MRI or CSF findings be maintained for PPMS, with replacement of the previous brain imaging criterion with the new MAGNIMS brain imaging criterion for DIS (2 of 3 of the following: ≥1 T2 lesions in at least 1 area characteristic for MS [periventricular, juxtacortical, or infratentorial]; ≥2 T2 lesions in the cord; or positive CSF [isoelectric focusing evidence of oligoclonal bands and/or elevated IgG index]) (Table 3). This consensus-based recommendation is justified by comparing diagnostic criteria for PPMS^36^ and by a subsequent reanalysis of these data (X. Montalban, personal communication). Use of MAGNIMS-based imaging criteria for PPMS with or without associated CSF evaluation should be supported by additional data further documenting the sensitivity and specificity of the criteria in this population.

**TABLE 3:** 2010 McDonald Criteria for Diagnosis of MS in Disease with Progression from Onset

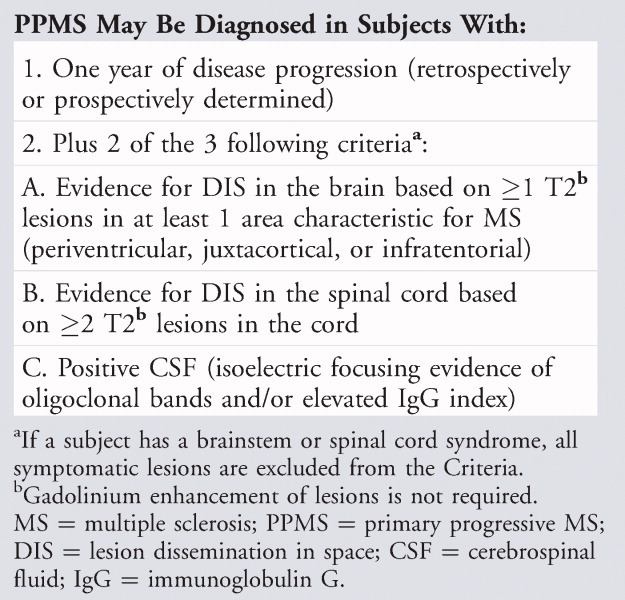

#### Applicability of the McDonald Criteria in Pediatric, Asian, and Latin American Populations

The McDonald Criteria were developed with data gathered largely from adult Caucasian European and North American populations, and their applicability has been questioned for other populations, particularly pediatric cases,^37^,^38^ Asians,^39^,^40^ and Latin Americans.^41^

### Pediatric MS

Over 95% of pediatric MS patients have an initial relapsing–remitting disease course, whereas PPMS is exceptional in children and should prompt detailed consideration of alternative diagnoses.^42^–^45^ About 80% of pediatric cases, and nearly all adolescent onset cases, present with attacks typical for adult CIS, with a similar or greater total T2 lesion burden.^46^–^48^ In children younger than 11 years, lesions are larger and more ill-defined than in teenagers.^49^ Imaging criteria for demonstrating DIS in pediatric MS show high sensitivity and/or specificity.^38^,^50^,^51^

The Panel's consensus was that the proposed MAGNIMS-based MRI revisions for DIS will also serve well for most pediatric MS patients, especially those with acute demyelination presenting as CIS, because most pediatric patients will have >2 lesions and are very likely to have lesions in 2 of the 4 specified CNS locations (periventricular, brainstem-infratentorial, juxtacortical, or spinal cord). The frequency of spinal cord lesions in pediatric MS patients is currently unreported, but the appearance of cord lesions in pediatric MS patients with spinal cord symptoms appears generally similar to that of adults.^52^

However, approximately 15 to 20% of pediatric MS patients, most aged <11 years, present with encephalopathy and multifocal neurological deficits difficult to distinguish from acute disseminated encephalomyelitis (ADEM).^43^,^50^ Current operational international consensus criteria for MS diagnosis in children with an ADEM-like first attack require confirmation by 2 or more non-ADEM like attacks, or 1 non-ADEM attack followed by accrual of clinically silent lesions.^53^ Although children with an ADEM-like first MS attack are more likely than children with monophasic ADEM to have 1 or more non-enhancing T1 hypointense lesions, 2 or more periventricular lesions, and the absence of a diffuse lesion pattern,^54^ these features are not absolutely discriminatory. Furthermore, MRI scans of children with monophasic ADEM typically demonstrate multiple variably enhancing lesions (often >2) typically located in the juxtacortical white matter, infratentorial space, and spinal cord. Thus, application of the revised MAGNIMS-based criteria for DIS and DIT on initial MRI would be inappropriate for such patients, and serial clinical and MRI observations are required to confirm a diagnosis of MS. In this young age group, there can be marked lesion resolution following an initial attack^49^ prior to emergence over time of new lesions and attacks leading to a diagnosis of MS.

### MS in Asian and Latin American Populations

Among Asian patients with CNS inflammatory demyelinating disease, a phenotype characterized by NMO, longitudinally extensive spinal cord lesions, and positive AQP4 autoantibody seropositivity^19^ has been relatively more common than in Western populations.^55^–^57^ The Panel solicited input on use of the McDonald Criteria in Asia and Latin America, where there is evidence of a similar phenotype distinction.^41^ Although the McDonald Criteria are widely used in these parts of the world, there is some uncertainty, especially in Asia, about whether MS and NMO are distinct and if so, how they should be distinguished.^39^ As currently applied, the term opticospinal MS appears to be an admixture of conventional MS and NMO. Confusion has arisen (1) because of the recognition that most cases of NMO are relapsing; (2) because AQP4 autoantibody testing has facilitated the diagnosis of NMO and permitted inclusion of individuals with symptomatic brain lesions who would previously have been excluded; and (3) because of the recognition that selective involvement of optic nerve and spinal cord alone does not differentiate NMO from MS.^58^ It is insufficient to make a diagnosis of NMO in the absence of the required specificity criteria of the revised Wingerchuk Criteria for “definite” NMO, which recommend presence of optic neuritis, acute myelitis, and at least 2 of 3 supportive paraclinical assessments (a contiguous spinal cord lesion at least 3 segments in length, brain MRI at onset that is nondiagnostic for MS, or NMO-IgG seropositivity).^59^ These criteria are successful in most instances to distinguish NMO from MS in patients with optic neuritis and myelitis, but the spectrum of NMO includes recurrent myelitis and optic neuritis, NMO syndromes with symptomatic brain lesions at presentation, and NMO associated with systemic autoimmune diseases.^60^ Failure to make the correct diagnosis in patients with NMO may impact treatment.^20^

The Panel recommends testing for AQP4 autoantibodies with validated assays in patients who are suspected of having NMO or NMO spectrum disorders, especially in patients with Asian or Latin American genetic background because of the higher prevalence of the disease in these populations. Such testing may be less important in those subjects presenting with conventional Western type MS. Although not all patients with an NMO-like presentation will be AQP4 antibody positive, the majority are, whereas those with MS are more likely to be AQP4 antibody negative.^16^,^56^,^61^ Current evidence suggests that once NMO and NMO spectrum disorders have been excluded, Western type MS in Asia or Latin America is not fundamentally different from typical MS in the Caucasian population, and that the MAGNIMS MRI criteria would apply for such patients, although confirmatory studies should be done.

### The McDonald Criteria: 2010 Revisions

#### Application of the Criteria

The Panel recommends revisions to the McDonald Criteria for diagnosis of MS (Table 4) focusing specifically on requirements to demonstrate DIS, DIT, and on diagnosis of PPMS. These 2010 revisions to the McDonald Criteria are likely to be applicable in pediatric, Asian, and Latin American populations once careful evaluation for other potential explanations for the clinical presentation is made. The predictive validity of DIS and DIT based on a single first scan in children with CIS needs to be confirmed in prospective studies. The McDonald Criteria have not yet been validated in Asian and Latin American populations, and studies need to be done to confirm the sensitivity and specificity of the Criteria in such patients. Care must be taken to exclude NMO as a differential diagnosis, which can be confounded by the imperfect sensitivity of AQP-4 autoantibody assays, the presence of brain lesions in NMO, and the difficulty of detecting long spinal cord lesions in immunosuppressed patients.

**TABLE 4:** The 2010 McDonald Criteria for Diagnosis of MS

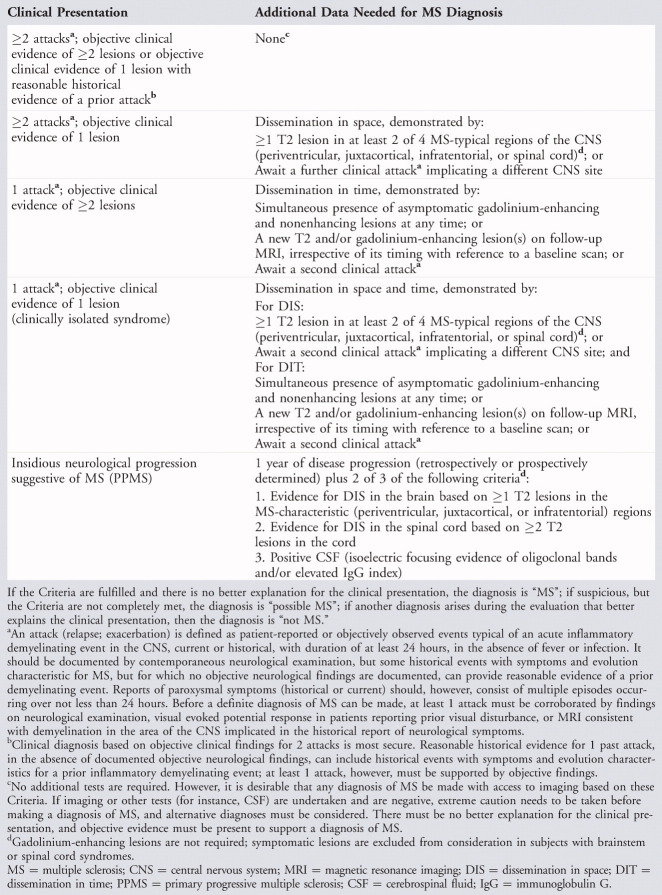

### Future Directions

#### Potential Added Value of Biomarkers

Although increased IgG index or the presence of oligoclonal bands in the CSF support an MS diagnosis, and AQP4 antibody assays can help in the differential diagnosis process, there are still no specific biomarkers to confirm the diagnosis. Several blood and CSF biomarkers may be promising,^62^–^65^ and high-resolution spectral domain optical coherence tomography might be as good as VEP in assessing visual involvement.^66^ The diagnostic utility of such markers needs to be validated and tested prospectively.

#### Refinements in Imaging Criteria

The McDonald Criteria were based on detection of lesions generally using 1.5T magnet strength in noncortical regions of the brain and spinal cord. However, a large proportion of MS lesions are in the cortex^67^,^68^ and can be detected using double inversion recovery imaging.^69^–^74^ The presence of at least 1 intracortical lesion in subjects with CIS may help identify subjects at high risk for developing CDMS.^75^ Magnet strengths >1.5T with tailored acquisition protocols^76^–^79^ may also enhance diagnosis, with improvements in image resolution, signal-to-noise ratio, and chemical shift. Scans at 7.0T showed lesions in the white and gray matter with enhanced in vivo detection of pathological hallmarks of MS lesions.^80^–^83^ Finally, MRI techniques such as magnetic transfer imaging allow the detection of damage outside focal lesions (for instance, in normal-appearing brain tissues) not present in conditions such as ADEM and NMO.^15^,^84^,^85^ The utility of these scanning technologies for MS diagnosis in patients with CIS remains a matter for future research and validation.

Many individuals with high lesion loads may have had a protracted subclinical disease course prior to their first clinical event. As a consequence, occasional individuals investigated by MRI for indications unrelated to MS have incidental findings of brain lesions with appearance and topography consistent with MS. Detection of this presymptomatic phase, or radiologically isolated syndrome, is increasingly common. Some of these individuals followed clinically and by serial imaging will develop DIT by MRI, and some have clinical disease-defining events after several years.^86^–^89^ However, in the absence of supportive research findings, the Panel concluded that a firm diagnosis of MS based on incidental findings on MRI alone, even with additional supportive findings on evoked potentials or typical CSF findings in the absence of MS-relevant clinical symptoms, is problematic. A future definite diagnosis of MS, however, cannot be excluded and may be likely, depending on the evolution of neurologic symptoms and signs.

### Conclusions

The 2010 revisions to the McDonald Criteria will in some instances allow a more rapid diagnosis of MS, with equivalent or improved specificity and/or sensitivity compared with past Criteria and will in many instances clarify and simplify the diagnostic process with fewer required MRI examinations. A proportion of patients with nonspecific symptoms (eg, fatigue, weakness, or dizziness) and nonspecific MRI findings are referred to secondary and tertiary MS centers in the developed world for a second opinion and do not in fact have MS.^90^ These revised McDonald Criteria for MS diagnosis should therefore be applied only when patients have experienced a typical CIS (or progressive paraparesis/cerebellar/cognitive syndrome in the case of suspected PPMS).

The Panel acknowledges that using these refined diagnostic criteria may change some of the outcomes of patients in natural history studies and clinical trials, when original expectations for outcomes may be based on subjects whose diagnosis was made using past, somewhat different criteria.^91^ Most of the currently recommended revisions are based upon new data generated since the 2005 revisions. However, there remains a need for further testing in prospective and retrospective datasets of many of these criteria, especially in populations of patients typical of those seen in general neurology practices, both to further assess their value and utility and to provide suggestions for further refinements in the future.

## Potential Conflicts of Interest

C.H.P.: consultancy, Actelion, Biogen Idec, Bayer Schering, Teva, Merck-Serono, Novartis, Glaxo SK, UCB, Roche, Antisense Ther; expert testimony, Biogen Idec; grants/grants pending, Biogen Idec, Bayer Schering, Teva, Merck-Serono, Novartis, Glaxo SK, UCB. S.C.R.: travel support, US NMSS, ECTRIMS, Multiple Sclerosis International Federation, MS Ireland; payment for writing or reviewing manuscript, US NMSS, ECTRIMS; consultancy, US NMSS, ECTRIMS, Sanofi-Aventis, Bayer Schering Pharma, BioMarin, EMD Merck Serono, Mt Sinai College of Medicine (New York, NY), European Committee for Treatment and Research in MS, Eisai, INC Research, Eli Lilly Inc, Isis Pharmaceuticals Inc, MediciNova, Cleveland Clinic Foundation, Free University Amsterdam, Genentech/F. Hoffmann-LaRoche, Synthon BV, Antisense Therapeutics Ltd, BaroFold, Protein Design Laboratories; royalties, Demos Medical Publishers (New York, NY). B.B.: travel support, US NMSS, ECTRIMS, MSIF, MS Ireland; consultancy, Biogen Idec, Genzyme; grants/grants pending, Multiple Sclerosis Society of Canada, Canadian Institutes of Health Research; paid educational presentations, honoraria for symposia at the American Academy of Neurology. M.C.: board membership, GENMAB; consultancy, Biogen, Genzyme; grants/grants pending, Bayer Schering, Biogen Elan, Novartis, Merck Serono, Sanofi-Aventis, Teva. J.A.C.: travel expenses, US NMSS; consultancy, Biogen Idec, Lilly, Novartis, Serono, Teva; grants/grants pending, Department of Defense, NIH, US NMSS; speaking fees, Biogen Idec, Novartis, Sanofi-Aventis, Waterfront Media. M.F.: travel expenses, US NMSS, ECTRIMS, MSIF, MS Ireland; board membership, Teva Pharmaceutical Industries Ltd, Genmab A/S; consultancy, Bayer Schering Pharma, Biogen-Dompé AG, Genmab A/S, Merck Serono, Pepgen Corporation, Teva Pharmaceutical Industries Ltd; grants/grants pending, Bayer-Schering, Biogen-Dompé AG, Genmab A/S, Merck Serono, Teva Pharmaceutical Industries Ltd, Fondazione Italiana Sclerosi Multipla, Fondazione Mariani; speaking fees, Bayer Schering Pharma, Biogen-Dompé AG, Genmab A/S, Merck Serono, Teva Pharmaceutical Industries Ltd; travel expenses, Teva, Biogen-Dompé AG, Merck-Serono, Sanofi-Aventis, Genmab, Bayer Schering. K.F.: travel expenses, US NMSS, ECTRIMS, MSIF, MS Ireland; consultancy, Bayer Schering Pharma, Biogen Idec, Merck Serono; grants/grants pending, Bayer Schering Pharma, Biogen Idec, Asahi Kasei Kuraray Medical Co Ltd, Chemo-Sero-Therapeutic Research Institute, Mitsubishi Tanabe Pharma, Teijin Pharma, Theva Pharmaceutical, Eisai Inc, Kowa Pharmaceutical, Ministry of Education, Science, and Technology of Japan, Ministry of Health, Labor, and Welfare of Japan; speaking fees, Bayer Schering Pharma, Biogen Idec, Eisai Inc, Mitsubishi Tanabe Pharma, Astellas Pharma, Takeda Pharmaceutical Company Ltd, Asahi Kasei Kuraray Medical Co; paid manuscript preparation, Cosmic Corporation; royalties, Bunkodo. E.H.: travel expenses, US NMSS, ECTRIMS, MSIF, MS Ireland; consultancy, Biogen Idec, Genzyme, Merck Serono, Novartis, Grifols; grants/grants pending, Biogen Idec; speaking fees, Biogen Idec, Genzyme, Merck Serono, Novartis, Bayer Healthcare, Sanofi-Aventis; paid educational presentations, Novartis. M.H.: consultancy, Biogen Idec; grant/grants pending, Health Research Board Ireland; speaking fees, Biogen Idec. L.K.: travel expenses, US NMSS; board membership, Editorial Board of Multiple Sclerosis; grants/grants pending, National Research Foundation Switzerland, Rubatto Foundation, Swiss MS society, European Union, Roche Foundation, Novartis Foundation; speaking fees, various companies involved in development of MS therapeutics; paid educational presentations, Neurostatus System for Standardized Neurological Assessment. F.D.L.: travel support, US NMSS; consultancy, Novartis, Bayer, Biogen Idec, EMD Serono, Genentech, Teva Neuroscience, Genmab, Medicinova, Actelion, Allozyne, Sanofi-Aventis, Acorda, Questcor, Avanir, Roche, Celgene, Abbott, Pfizer, Morphosys; grants/grants pending, NIH, NMSS, Acorda, Biogen Idec, Teva, Novartis, Sanofi-Aventis; speaking fees, Genzyme, Teva, EMD Serono; paid educational presentations, various continuing medical education services; stock/stock options, cognition pharmaceuticals. X.M.: travel expenses, US NMSS; consultancy, Bayer Schering Pharma, Biogen Idec, EMD Merck Serono, Genentech, Genzyme, Novartis, Sanofi-Aventis, Teva Pharmaceuticals, Almirall; grants/grants pending, Bayer Schering Pharma, Biogen Idec, EMD Merck Serono, Genentech, Genzyme, Novartis, Sanofi-Aventis, Teva Pharmaceuticals, Almirall; speaking fees, Bayer Schering Pharma, Biogen Idec, EMD Merck Serono, Genentech, Genzyme, Novartis, Sanofi-Aventis, Teva Pharmaceuticals, Almirall. M.S.-W.: travel support, US NMSS, ECTRIMS, MSIF, MS Ireland; fees for review activities, Genentech, Merck Serono, Roche; board membership, Board of Directors of Active Biotech, Sweden; consultancy, Elan, Merck Serono; speaking fees, Bayer Health Care, Merck Serono, Serono Symposia International Foundation, Sanofi-Aventis, Swedish Bank SEB. A.J.T.: travel support, US NMSS, ECTRIMS, MSIF, MS Ireland; board membership, National Hospital Development Foundation, Patrick Berthoud Charitable Trust; consultancy, Weleda AG/Society for Clinical Research, Medical Research Council, MS Society of Great Britain, Merck Serono, Biogen Idec, DIGNA Biotech, Novartis, Eisai London Research Laboratories, Teva Pharmaceuticals; grants/grants pending, National Institute for Health Research, MS Society of Great Britain; speaking fees, Serono Symposia, Sanofi-Aventis; travel expenses, MS International Federation, US NMSS, Biogen Idec; honoraria, Editor-in-Chief of Multiple Sclerosis. E.W.: consultancy, Roche, Actelion; grants/grants pending, US NMSS, NIH; speaking fees, Teva; received free drug for a trial given by Sanofi-Aventis and Biogen Idec. B.W.: travel support, US NMSS; European Committee for Treatment of MS; MS International Foundation; MS Ireland; consultancy, Novartis, Biogen Idec; employment, Mayo Clinic; royalties, RSR Ltd. J.S.W.: travel support, US NMSS, ECTRIMS, MSIF, MS Ireland; board membership, Antisense Therapeutics Ltd, BCDecker, Novartis Pharmaceuticals, Sanofi-Aventis, Teva Pharmaceuticals, Eli Lilly, UCB; consultancy, Genentech, Novartis Pharmaceuticals, Sanofi-Aventis, Teva Neuroscience, Teva Pharmaceuticals, Acorda, Acetilon, Bayer HealthCare, Facet Biotech, Peptimmune; grants/grants pending, NIH, Sanofi-Aventis, Clayton Foundation for Research, US NMSS; honoraria for lectures, Consortium MS Centers, Sanofi-Aventis New Zealand, Sterling Meeting Services, USF Health Professionals, Texas Neurological Society, Teva Pharmaceuticals, Lone Star Chapter NMSS, ICHE, Pfizer EMD Serono, SUNY, Stony Brook Foundation, UTMB, Medscape CME, University of Buffalo, Serono Symposia International Foundation, University of Utah; royalties, Millipore (Chemicon International) Corporation.
